# Same, same but different: Exploring *Plasmodium* cell division during liver stage development

**DOI:** 10.1371/journal.ppat.1011210

**Published:** 2023-03-30

**Authors:** Magali Roques, Annina Bindschedler, Raphael Beyeler, Volker T. Heussler

**Affiliations:** 1 Institute of Cell Biology, University of Bern, Bern, Switzerland; 2 Graduate School for Cellular and Biomedical Sciences, University of Bern, Bern, Switzerland; Joan and Sanford I Weill Medical College of Cornell University, UNITED STATES

## Abstract

*Plasmodium* parasites have a complex life cycle alternating between a mosquito and a vertebrate host. Following the bite of an *Anopheles* female mosquito, *Plasmodium* sporozoites are transmitted from the skin to the liver; their first place of replication within the host. Successfully invaded sporozoites undergo a massive replication and growth involving asynchronous DNA replication and division that results in the generation of tens of thousands or even hundreds of thousands of merozoites depending on the *Plasmodium* species. The generation of a high number of daughter parasites requires biogenesis and segregation of organelles to finally reach a relatively synchronous cytokinesis event. At the end of liver stage (LS) development, merozoites are packed into merosomes and released into the bloodstream. They are then liberated and infect red blood cells to again produce merozoites by schizogony for the erythrocytic stage of the life cycle. Although parasite LS and asexual blood stage (ABS) differ in many respects, important similarities exist between the two. This review focuses on the cell division of *Plasmodium* parasite LS in comparison with other life cycle stages especially the parasite blood stage.

## Introduction

**Fig 1:**
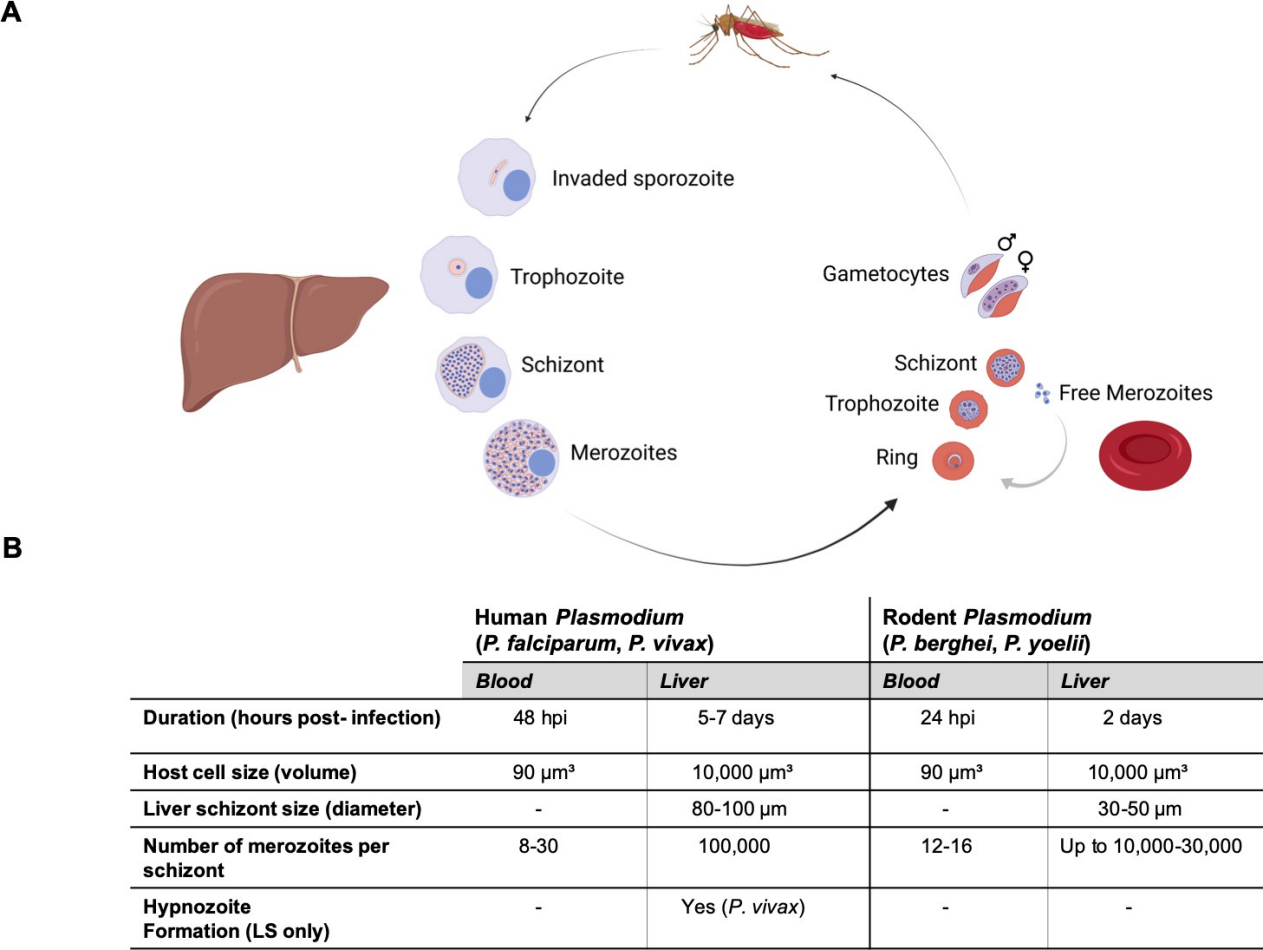
Replication of human and rodent *Plasmodium* parasites during exoerythrocytic and erythrocytic development. **(A)** *Plasmodium* life cycle with emphasis on the exoerythrocytic (liver) and erythrocytic (red blood cell) stages within the vertebrate host. Parasite development in the mosquito is symbolized by the depicted mosquito but is not shown. In the scheme, important exoerythrocytic (invaded sporozoite, trophozoite, schizont, and merozoites) and erythrocytic stages (ring, trophozoite, schizont, free merozoites, and gametocytes) are indicated (created with BioRender.com). **(B)** The table summarizes the main characteristics and morphological features of human (*P*. *falciparum*, *P*. *vivax*) and rodent (*P*. *berghei*, *P*. *yoelii*) *Plasmodium* parasites. Information is from [36,89,90,126] and also referred in the 2.2.2 section. LS, liver stage.

The genomes of different *Plasmodium* species contain about 5,000 genes and thus represent some of the most reduced genomes among eukaryotes (https://plasmodb.org/plasmo/app). To put this into context, the human genome, with about 30,000 genes, is 6 times larger. Even with such a reduced genome, the parasite must fulfill all basic requirements of a living cell, including cell division. It is therefore not surprising that genes related to eukaryotic cell cycle progression are conserved in the *Plasmodium* genome. Nevertheless, cell division in *Plasmodium* has some particularities suggesting that the parasite might also have genes that differ from other eukaryotes. Upon invasion of erythrocytes and hepatocytes, *Plasmodium* parasites initially grow without replicating their nuclei (called the trophozoite stage). After this phase, the parasite divides its nuclei without immediate cytokinesis and this results in the generation of multinucleated parasites called schizonts (Fig 1). Cytokinesis only happens at the end of schizogony and has been extensively reviewed for the blood stage of *Plasmodium* [1–3]. Although it has been speculated that parasite schizogony and merozoite formation in both hepatocytes and erythrocytes share some common gene regulatory networks and cell regulatory mechanisms and this needs to be proven [4–8]. Genetic manipulation of the parasite during its liver stage (LS) remains difficult due to: (1) the need for maintaining the entire parasite life cycle in the laboratory; (2) the limits of a conditional gene knockdown system during this phase; and (3) the low sporozoite–host cell infection rate. However, recent advances in microscopy, transcriptomics, proteomics, and high-throughput reverse genetic screens have allowed us to get a first idea about similarities and differences of the various replicative parasite stages including the LS [6,9–12]. Apart from the obvious similarities between LS and asexual blood stage (ABS) merozoite generation, there are also important differences. Exoerythrocytic schizogony results in thousands of nuclei outnumbering erythrocytic schizogony by more than 3 orders of magnitude (Fig 1) [5,6,8]. We therefore hypothesize that *Plasmodium* has evolved unique mechanisms that control its progression through the LS.

Although a basic set of genes involved in cell division are conserved in *Plasmodium* parasites, homologues of typical cell cycle checkpoint genes are entirely missing [13]. At a population level, this indeed makes sense as protist parasites rely on a relatively high mutation rate to escape host defense measures [14]. Cell cycle checkpoints that are important to guarantee correct DNA replication would thus be a disadvantage. Cell cycle-related genes are in general less numerous in *Plasmodium* parasites compared to, for example, humans (https://plasmodb.org/plasmo/app; [15]). One reason might be the way *Plasmodium* parasites divide their nuclei. Like yeast, they use a so-called “closed mitosis,” in which the nuclear membrane is not resolved [16]. This allows components involved in nuclear membrane assembly and disassembly to be excluded and thus saves time and resources.

Understanding the peculiarities of the molecular basis of cell division in *Plasmodium* LS cell division might open new avenues to control infection at an early, nonpathogenic stage.

In this review, we summarize what is known to date about *Plasmodium* LS cell division and the development from sporozoite invasion into the hepatocyte to daughter merozoite formation, and we discuss the mechanisms and players involved in these events. In particular, we will point out the similarities and differences of *Plasmodium* ABS and LS cell division as merozoites capable of infecting red blood cells are formed at the end of both developmental stages.

## 1) Triggering parasite development: From the transmissive sporozoite to the exoerythrocytic trophozoite stage

**Fig 2:**
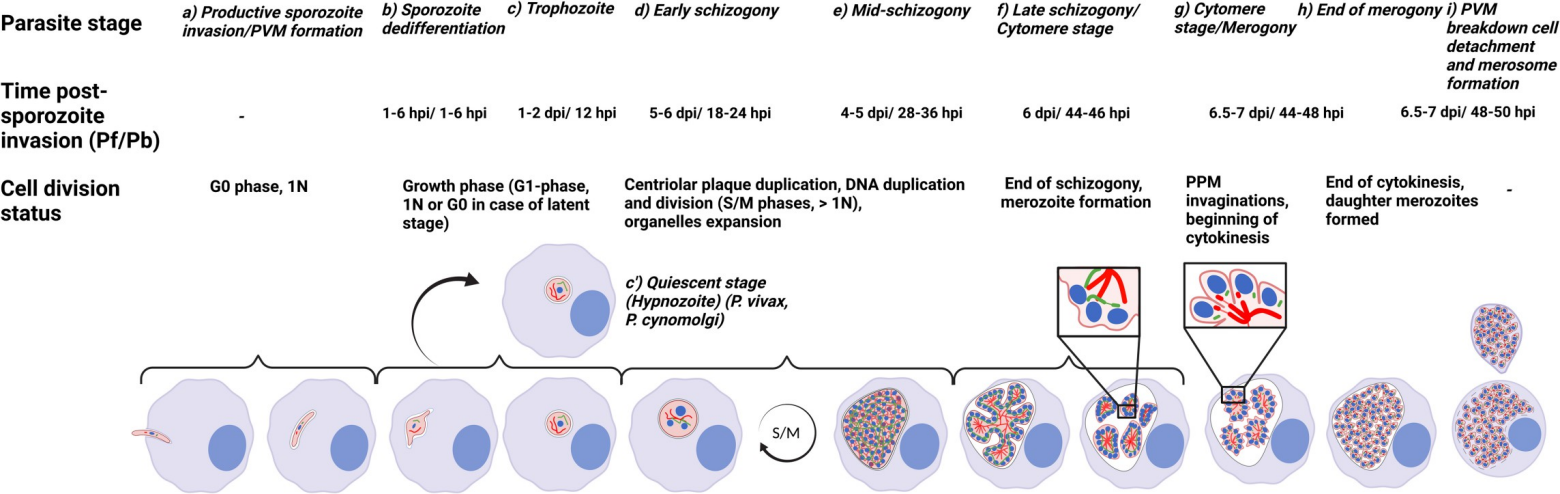
Schematic representation of *Plasmodium* cell division and development during the LS. Typical features and molecular details of *P*. *falciparum* and *P*. *berghei* (*Pf*/*Pb*) during the entire LS development are indicated as “Parasite stage” from (a) to (i) and the “Cell division status” section are indicated for each parasite stage. The duration of development “Time post-sporozoite invasion (Pf/Pb)” indicates the in vivo *Plasmodium* LS development. Horizontal brackets assemble drawings that represent the “Cell division status” of the parasite. **(a)** Upon productive invasion of a hepatocyte (light blue) the parasite (pink) resides within a PV surrounded by the PVM (light blue). Initially, the parasite remains in G0, 1N but expands in size. Parasite organelles are depicted as follows: nucleus (blue), apicoplast (green), and mitochondria (red). **(b)** The dedifferentiation phase starts when the crescent-shape sporozoite transforms into a bulbous-shaped form that finally becomes a circular EEF trophozoite expanding in size and entering the G1 phase **(c)**. **(c’)** A proportion of *P*. *vivax* and *P*. *cynomolgi* parasites do not develop further into schizonts but remain dormant (hypnozoite) as in G0 phase. **(d)** Schizogony is characterized by nuclei multiplication, and alternated DNA synthesis/mitosis (S/M) phases are triggered (DNA >1N). **(e)** During the process of schizogony, mitochondrion and apicoplast grow into highly branched structures. **(f)** The cytomere stage initiates merogony (merozoite formation). The PPM invaginates and the still singular mitochondrion organizes as a finger-like structure. The apicoplast locates between the nuclei and the surrounding PPM within parasite cytoplasm (left panel in **(f)**). Then, the apicoplast begins to form regular constrictions resulting in the fission of the organelle (right panel in **(f)**). **(g)** At the end of the cytomere stage, mitochondrion fission occurs prior to cytokinesis as depicted in the zoomed section. **(h)** Tens of thousands individual merozoites are formed, depending on the *Plasmodium* species, with each containing an individual set of organelles; cytokinesis signifies the end of LS merogony. **(i)** Finally, PVM rupture liberates merozoites into the host cytosol followed by host cell detachment and merosome formation (indicated by the merozoite-filled vesicle on top of the detached cell (created with BioRender.com). EEF, exoerythrocytic form; LS, liver stage; PPM, parasite plasma membrane; PVM, parasitophorous vacuole membrane.

**Fig 3:**
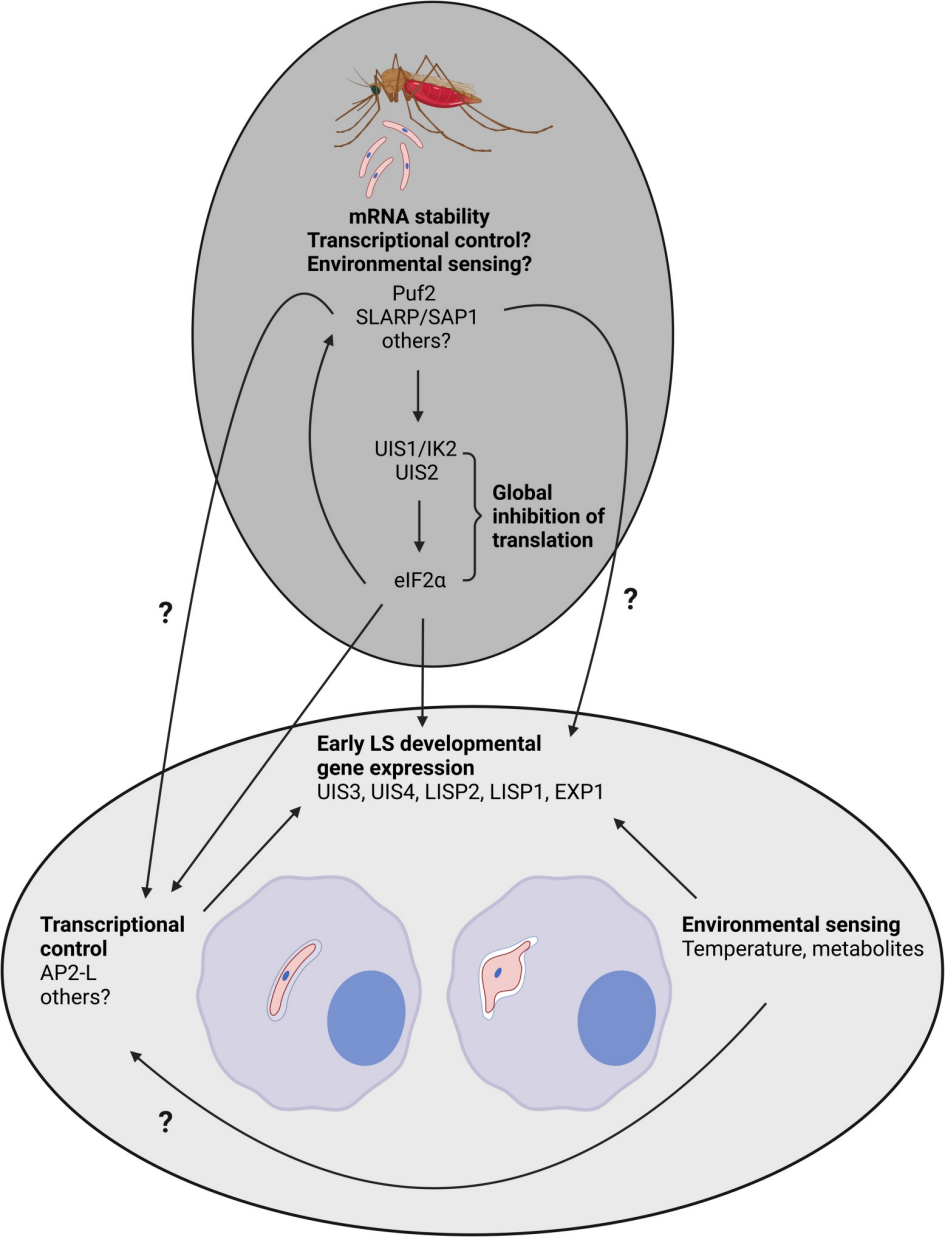
Parasite and host cell factors involved in sporozoite transformation into EEF represented as hierarchical events from mosquito to LS. The dark gray circle summarizes parasite factors currently known to exert posttranscriptional and translational control on sporozoites within mosquito salivary glands. The light gray circle summarizes parasite and host cell factors shown to have an effect on sporozoite transformation into EEF within the host hepatocyte. Arrows indicate the hierarchical events happening from sporozoite latency in the mosquito to the early LS developmental gene expression within hepatocyte. Question marks show a hypothetical role of factors acting directly on the sporozoite to EEF transition. References are listed in Table 1: pumilio-2 (Puf2), sporozoite and LS asparagine-rich protein/sporozoite asparagine-rich protein (SLARP/SAP1), up-regulated in infective sporozoites 1/initiation factor 2 kinase (UIS1/IK2), up-regulated in infective sporozoites 2 (UIS2), eukaryotic translation initiation factor 2 subunit alpha (eIF2α), AP2 domain transcription factor (AP2-L), liver-specific protein 2 (LISP2) and here for up-regulated in infective sporozoite 3 (UIS3) [127], up-regulated in infective sporozoite 4 (UIS4) [67], and here for liver-specific protein 1 (LISP1) [128], exported protein 1 (EXP1) [129], temperature and metabolites [28–30] (created with BioRender.com). EEF, exoerythrocytic form; LS, liver stage.

In mosquito and vertebrate hosts, *Plasmodium* parasites experience different physiological environments that require a rapid molecular and cellular reprogramming during host switch. In this section, we explore the triggering signals that activate the transition between the nondividing sporozoite stage and the beginning of the exoerythrocytic form (EEF) development called the trophozoite stage (Fig 1A). Posttranscriptional/translational controls and environmental changes (temperature, pH, nutrients) act together as regulators of gene expression by activating various signaling networks that enable parasites to sense and adapt to varying environmental conditions (Figs 2A–2C and 3).

### 1.1 Posttranscriptional and translational controls in the latent sporozoite stage

Developmental arrest of the sporozoite within the mosquito salivary glands is due to the stability of the mRNA repertoire during this stage. Neither transcription nor mRNA degradation have been detected within this latent phase. Rather, mRNAs remain ready for just-in-time translation at the next stage upon being transmitted to the mammal host, as reviewed previously [17,18]. In salivary gland-derived sporozoites, like in gametocytes, this process is tightly controlled by 2 mechanisms: the translational hierarchy and posttranscriptional silencing (Table 1 and Figs 2A and 2B and 3).

**Table 1:** List of proteins involved in *Plasmodium* LS cell division and development mentioned within this review.

Name	Full name	*P*. *falciparum* PF3D7_ID	*P*. *berghei* PBANKA_ID	Blood screen* = Relative growth rate	M-L screen** = Log_2_-fold reduction	Function during *Plasmodium* LS division/development
From sporozoite to EEF transformation/trophozoite						
PUF2	Pumilio-2	0417100	0719200	No information	No information	mRNA-binding protein, posttranscriptional inhibition in SG sporozoites (*Pb*) [22–24]
SLARP/ SAP1	Sporozoite and LS asparagine-rich protein/sporozoite asparagine-rich protein	1147000	0902100	0.92, Dispensable	−4.97, Reduced	Maintenance of mRNA in salivary gland sporozoites (*Py*, *Pb*) [25–27]
eIF2α	Eukaryotic translation initiation factor 2 subunit alpha	0728000	0212100	No information	No information	Translational inhibition in salivary gland sporozoites (*Pb*) [19]
UIS1/IK2	Up-regulated in infective sporozoites 1/initiation factor 2 kinase	0107600	0205800	0.25 Slow	5.9 Not Reduced	Serine/threonine protein kinase; translational inhibition in salivary gland sporozoites (*Pb*) [19]
UIS2	Up-regulated in infective sporozoites 2	1464600	1328000	0.10 Essential	No information	eIF2α phosphatase; translational inhibition in SG sporozoites (*Pb*) [21]
From Trophozoite to Merozoite formation						
AP2-L	AP2 domain transcription factor	0730300	0214400	0.71, Slow	−6.62, Reduced	Transcription regulation (*Pb*) [75]
PlasMei2	Plasmodium Meiosis inhibited 2 RNA-binding protein	0623400	1122300	1.05, Dispensable	−10.53, Reduced	Schizogony progression (*Pf*, *Py*) [37,49]
LISP2	Liver-specific protein 2	0405300	1003000	No information	No information	Schizogony, merogony completion (*Pb*, *Pv*) [68]
SPELD	Sporozoite surface protein essential for LS development	1137800	0910900	No information	No information	Schizogony, merogony completion (*Pb*) [69]
PALM	Plasmodium apicoplast protein important for liver merozoite formation	0602300	0101100	1.00, Dispensable	−4.93, Reduced	Merozoite segregation (*Pb*) [111]
ATG8	Autophagy-related protein 8	1019900	0504100	0.07, Essential	No information	Apicoplast maintenance (*P*b) [81]

*From *P*. *berghei* ABS high-throughput screen [42].

**From *P*. *bergei* mosquito and liver screen [10].

*Plasmodium* proteins with a confirmed or putative role in LS parasite cell division and development. Parasite protein names and *P*. *falciparum* and *P*. *berghei* ID number are listed. High-throughput screen data of ABS [42] and M-L [10] are provided. Information on their function during LS is based on reverse genetic studies. No information means that no screen data were available (no vector available at that moment of the study or blood stage essential). ABS, asexual blood stage; EEF, exoerythrocytic form; LS, liver stage.

It has been shown that the global inhibition of protein synthesis in sporozoites is mediated through the phosphorylation of the translational eukaryotic initiation factor 2α (eIF2α) by the protein kinase IK2 also named “up-regulated in infectious sporozoites 1” (UIS1) [19,20]. *ik2/uis1-*deficient sporozoites showed a reduced infectivity associated with the premature transformation of sporozoites into EEF within the mosquito salivary glands [19]. Dephosphorylation of eIF2α is regulated by the essential phosphatase “up-regulated in infectious sporozoites 2” (UIS2), which is expressed in salivary gland sporozoites and blood stages, and the conditional expression of *Pbuis2* showed a defect in LS development within the mutant parasites associated with an increased level of phosphorylation of eIF2α [21]. This suggests a possible role of UIS2 in promoting the reactivation of translation once the sporozoite has entered the mammalian host allowing sporozoite transformation and LS development. Still, it remains to be shown how UIS2 itself is regulated.

The second mechanism of gene expression control employed by *Plasmodium* sporozoites is translational silencing. The storage of translationally silent mRNA avoids the premature expression of LS-specific proteins. It is mediated by the RNA-binding protein (RBP), Pumilio and fem-3binding factor homology 2 (Puf2) (Table 1 and Figs 2A and 2B and 3). Interestingly, *puf2*-deficient *P*. *berghei* sporozoites can still invade hepatocytes early after they have invaded the salivary glands [22,23]. However, Puf2 remains essential for the maintenance of sporozoite infectivity during prolonged periods within mosquito salivary glands. Moreover, over time, *Puf2*-deficient sporozoites prematurely transform into round forms resembling EEF and lose their infectivity [22–24].

A similar phenotype of premature transformation has also been observed for parasites deficient for the *ik2* gene that is controlled by Puf2 [19]. Thus, Puf2 appears to be a central player in mRNA silencing in *Plasmodium* EEFs. In addition to UIS2 and IK2, Puf2 appears to control translation of mRNA coding for proteins involved in the *Plasmodium* early liver developmental stage such as UIS2, UIS3, UIS4, exported protein 1 (EXP1), liver-specific protein 2 (LISP2), which are all proteins involved in parasitophorous vacuole membrane (PVM) remodeling post-sporozoite invasion of hepatocytes [22–24].

The sporozoite and LS asparagine-rich protein or sporozoite asparagine-rich protein (SLARP/SAP1) has been shown to be involved in LS-specific transcript storage and stabilization in the sporozoite of *P*. *berghei* [25] and *P*. *yoelii* [26,27] (Figs 2B and 3). Interestingly, SLARP/SAP1 is conserved among *Plasmodium* species and is considered to be one of the major regulators of early LS development as the deletion of this gene results in reduced expression of essential early LS developmental genes (e.g., *uis3*, *uis4*) within *P*. *berghei* and *P*. *yoelii* [25–27]. This is coherent with the phenotype observed for *slarp/sap1*-deleted parasites as the EEFs remain small, uninucleated and fail to replicate further [25,26]. SLARP/SAP1 plays an essential role in mRNA stabilization within sporozoites in the salivary glands but the mechanism of regulation remains unknown [25–27]. The reported discrepancy of SLARP/SAP1 localization in 2 rodent *Plasmodium* parasites—cytoplasmic in *P*. *yoelii* salivary gland sporozoites [26,27] and nuclear in *P*. *berghei* salivary gland sporozoites and EEF [25]—remains puzzling. The fact that SLARP does not contain any known RNA-binding functional domain suggests alternative transcript stabilization mechanisms that remain to be defined.

### 1.2 Environmental changes trigger *Plasmodium* LS development

Transformation of the crescent-shape sporozoite into a premature circular EEF can be observed in vitro when sporozoites are cultured in L15 cell culture medium containing fetal calf serum (axenic culture conditions, Figs 2B and 3). This suggests that the sporozoite can sense its environment, similar to gametocytes that have been ingested by mosquitoes during a blood meal. Indeed, it has been shown that cell culture medium, a rise in temperature (from 22°C to 37°C), bicarbonate, serum, and glucose metabolites are needed for the dedifferentiation process [28–30]. Transformation triggered by medium and a temperature shift results in an increase in intracellular calcium localizing at the parasite spherical bulb [31]. Nevertheless, *Plasmodium* LS axenic cultivated sporozoites failed to replicate and are thus not able to enter LS schizogony [28–30].

We therefore conclude that environmental sensing and posttranscriptional controls are intimately linked and cooperate to trigger the transformation from sporozoite to growing trophozoite.

## 2) Time to divide! From the trophozoite stage to the merozoite stage

**Fig 4:**
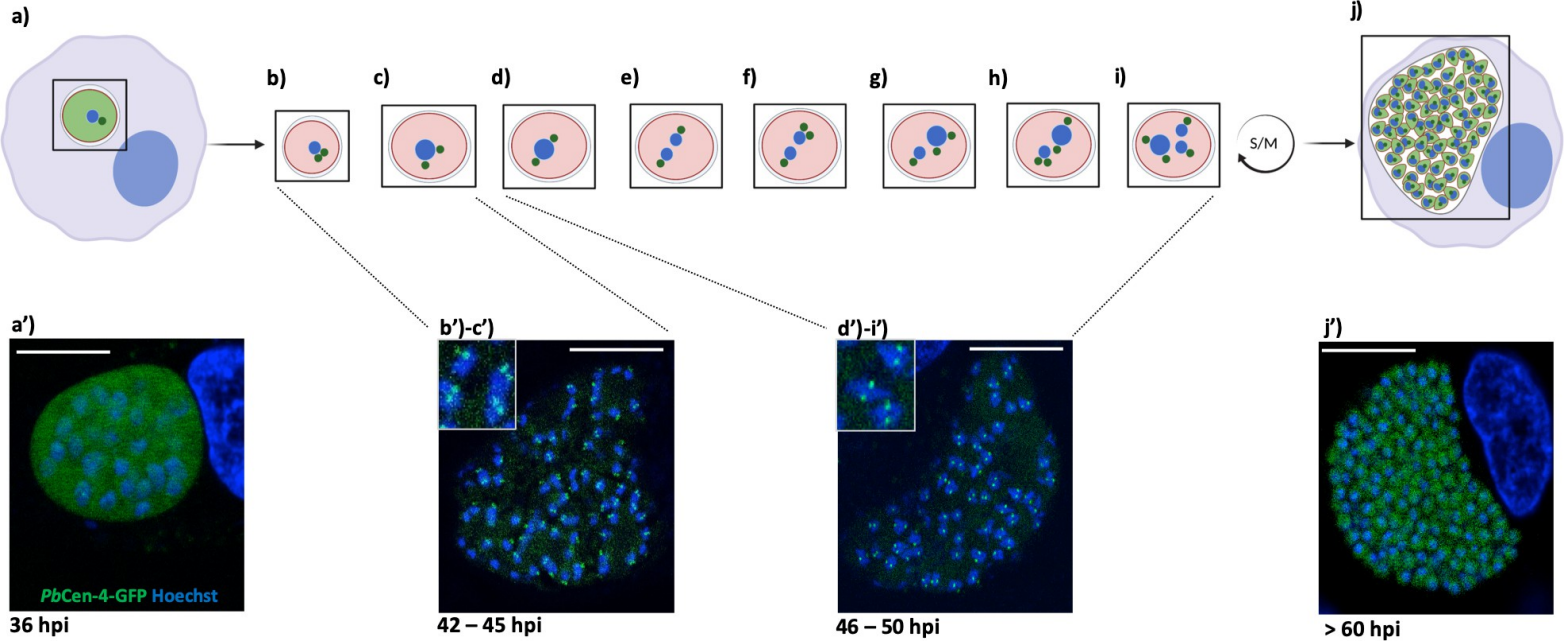
Schematic representation of CPs with *Pb*Cen-4-GFP and nucleus behavior during LS *Plasmodium* schizogony. The scheme from **(a)** to **(j)** represents *Plasmodium* nuclear and CP duplication and division through LS schizogony. Individual intracellular *Plasmodium* EEF (pink, from **(b)** to **(i)**; green light in **(a)** and **(j)**) surrounded by the PVM (light gray) within a hepatocyte (light blue) and the parasite CP (dark green) that is represented as a dot proximal to the parasite nucleus (blue). Corresponding live cell confocal images to this scheme represent prominent *Plasmodium* LS developmental stages (hpi: hours post-sporozoite infection into host cell): as early schizont **(a’)**, progression of schizogony (mid-schizogony) from **(b’)** to **(c’)**, late schizogony/beginning of cytomere stage from **(d’) to (i’)**, and finally end of merogony (merozoites formation) **(j’)**. These images represent endogenous tagging of *Pb*Cen-4 (PBANKA_0941400, CP marker) in fusion with a GFP fluorescent marker at its C-terminus (GFP, green) during LS development. Parasite and host cell nucleus are stained with Hoechst 33342 (blue). The transgenic parasite line has been generated previously [8]. HeLa cells were infected with *Pb*Cen-4-GFP expressing salivary gland sporozoites. Images represent the progression of *Plasmodiu*m nuclear division through schizogony. For a better comprehension, 2 S/M asynchronous rounds where an early schizont stage duplicates its CP have been presented (b); the 2 CPs move at the opposite part of the starting duplicating nucleus (c), (d), from (b’) to (c’) and from (d’) to (i’) to finally be present as a dot close to each individual separated nuclei (e). The S/M alternative phases (represented by the arrow) are then repeated from (f) to (i) (second round of S/M represented here) until cytomere stage formation and merozoite formation in (j) and (j’). The scale bar for the early schizont image (a’) is 10 μm and for the late schizonts (from (b’) to (c’); from (d’) to (i’)) and end of merozoites formation (j’) is 20 μm (created with BioRender.com). CP, centriolar plaque; EEF, exoerythrocytic form; LS, liver stage; PVM, parasitophorous vacuole membrane.

Liver stage schizogony starts at the transition of trophozoite to early schizont where the parasite commences its DNA replication and nuclear division (karyokinesis) begins. Organelles, like mitochondria and apicoplast, expand generating huge, intertwined networks, and membrane production accelerates to a maximum when parasites reach the cytomere stage. During the cytomere stage, the individual nuclei and a set of other essential organelles are packed within the newly formed parasite plasma membrane (PPM) and separate from each other by cytokinesis to finalize the formation of exoerythrocytic merozoites ready to invade red blood cells. The cellular and molecular aspects involved in karyokinesis, organellar/membrane biogenesis and segregation and cytokinesis occurring within *Plasmodium* LS are discussed in the current section (Table 1 and Figs 2D–2I and 4).

### 2.1 Karyokinesis in *Plasmodium* LS

#### 2.1.1 *Plasmodium* LS growth: In vitro versus in vivo

After rounding up, the trophozoite proceeds to duplicate its genome to distribute genetic material to its progeny. Advanced microscopy recently showed that the cellular mechanism of LS karyokinesis is similar to ABS karyokinesis but is performed at a much larger scale (Fig 1B; [6,8,32]). It is not known which factors causes this difference, but it can be speculated that it is again dependent on external factors [33–36]. A possible reason for this is that LS parasites grown in vivo are considerably larger than those cultivated in vitro [36,37]. There is also a size difference depending on the cell type used for in vitro culture, whereby parasites grow significantly larger in primary hepatocytes as compared to hepatoma cells, supporting the hypothesis that environmental factors are critical for parasite growth. Nutrient restriction might be a prime factor and some studies clearly point in this direction for ABS parasite growth [38,39], but it remains to be proven for LS parasites at an experimental level [35,36]. Another simple explanation is that primary hepatocytes in vivo might be more flexible in their expansion compared to cells cultivated in vitro. It is known that the parasite can expand its host cell considerably in vivo to sizes several times that of a normal hepatocyte, a phenomenon that has never been observed in vitro. This observation can possibly be addressed using liver organoids in microfluidics systems that closely emulate physiological conditions.

Genetic and time factors might also contribute to the differences in growth of LS parasites. The fact that parasites residing for extended times in host cells cultivated in vitro can undergo a kind of programmed cell death resembling autophagic cell death [40,41] strongly supports time as a restriction factor. For ABS parasites, proteins such as Aurora kinases (e.g., Ark1), CDKs-related enzymes (Crks) (e.g., Crk4), never in mitosis (NIMA) kinases (e.g., Nek1), and cyclin (e.g., Cyc1) appear to be essential players for cell cycle progression [42–48]. The study of these proteins during parasite LS schizogony remains difficult in the absence of a robust conditional knockdown system, as reviewed previously [13,42]. Nevertheless, *Plasmodium* possesses proteins that are specifically expressed during its LS and these might play a role within the schizogony event [37,49]. Advances in genetic tools like promotor swap and other conditional technologies might help in the future to shed some light on this very important question of how the LS parasites control their growth [50–53].

#### 2.1.2 The asynchronous *Plasmodium* schizogony

The typical eukaryotic cell cycle is divided into controlled phases of cell growth (G1-phase), DNA replication (S-phase), preparation for mitosis (G2-phase) and nucleus division with DNA segregation, mitosis (M-phase). Usually, mitosis is followed immediately by cytokinesis where organelles and the cell cytosolic content are physically separated into 2 new daughter cells. To ensure proper division, a cell has to control each phase, and this is tightly coordinated via checkpoints [54]. In rodent *Plasmodium* parasites, the asynchronous exoerythrocytic schizogony starts around 18 to 24 hpi when the growing mature trophozoite (G1-phase, 1N) enters schizogony; this is characterized by multiple rounds of S- and M- phases (S/M alternated phases, >1N) generating thousands of nuclei without immediate cytokinesis after each S/M round (Figs 2D and 2E and 4A–4J) [1–3]. Schizogony progresses without nuclear membrane rupture as closed mitosis, a phenomenon also observed in yeast. After the last synchronous round of S/M, the PPM starts to invaginate forming typical clusters of nuclei and generating the cytomere stage (Fig 2F) [6,55,56]. Plasma membrane invagination continues until every nucleus and a set of essential organelles is completely separated and exoerythrocytic invasive merozoites are formed. This happens approximately 44 hpi in vitro, however, merozoite formation can be significantly faster in vivo (Fig 2F–2H) [6,8,56]. Importantly and in contrast to most other eukaryotic cells, due to the oscillating S/M-phases, it is assumed that *Plasmodium* schizogony lacks a G2-phase. In addition, no orthologs of conventional cell cycle checkpoints, like p53, ATM, ATR, and Rb, exist in *Plasmodium* [13].

In most eukaryotic cells, cell cycle control and progression through each phase is driven by cyclin-dependent kinases (CDKs) and their cyclin partners [54]. The parasite possesses an unusual set of CDK-like kinases (protein kinases (PKs) and CDKs-related enzymes (Crks)) and cyclins that have mainly been investigated in *Plasmodium* blood and mosquito stages, as reviewed previously [13]. None of the *Plasmodium* cyclins are phylogenetically clustered with the classical G1, S-phase, or mitotic cyclins from plants or animals. Even more importantly, they are not expressed in a cyclic manner during the *Plasmodium* cell cycle [13,46,47,57–59]. To date, no functional studies have been performed on CDK-like kinases and cyclins within the LS due to the essentiality of most of them for parasite survival in blood and mosquito stages [13,42]. Although transcriptomic studies confirmed expression of CDK-like kinases in *Plasmodium* LS [9], a possible role at this stage remains to be proven.

To date, little is known about the function of cyclin-dependent kinase regulatory subunit (CDKrs) proteins that act as both activator and inhibitor of CDK activity by functioning as docking factors for the CDK/cyclin substrate complexes in *Plasmodium* [60]. So far, in *P*. *berghei*, one CDKrs (PBANKA_0824400) is a binding partner of *Pb*Crk5 and has a role in microgametogenesis [57]. Interestingly, in addition to its function in *Plasmodium* sexual development, a recent large-scale reverse genetics screen revealed that PBANKA_0824400 gene deletion affects the parasite liver phase development without affecting the blood stage progression [10,42]. However, the exact role of this protein in LS schizogony progression remains to be defined.

In contrast to other eukaryotic cells, *Plasmodium* has no typical centrosomes/centrioles acting as nuclear microtubule organizing centers (MTOCs) to coordinate chromosome separation [61,62]. Instead, *Plasmodium* MTOC is referred to as the centriolar plaque (CP) and until recently was thought to be embedded within the nuclear membrane as spindle pole bodies (SPBs) based on studies in yeast [61–63]. However, recent studies using ultrastructure expansion microscopy (U-ExM) for *Pf*ABS demonstrated that the CP is not embedded within the nuclear envelope but remains extranuclear proximal to a nuclear pore marker during mitosis [32]. Whether this configuration is maintained during parasite LS division remains to be defined. As in mammalian centrosomes, *Plasmodium* CPs duplicate prior to DNA replication/division and execution of this event involves the concerted action of several kinases such as CDKs and centrins [13,64]. Several studies have identified CPs throughout the *Plasmodium* life cycle, mainly with antibodies detecting centrins (*Pf*Cen-1 and *Pf*Cen-3), and with a *P*. *berghei* line expressing *Pb*Cen-4-GFP [8,32,62,65]. In fact, this parasite line allowed the detection of a CP during *P*. *berghei* LS (Fig 4) [8]. When nuclear division has just started (early schizogony) or is completed, *Pb*Cen-4-GFP localizes as a faint yet discrete dot proximal to nuclei and is predominantly present within the parasite cytosol (Fig 4A and 4A’ and 4J and 4J’). In contrast, during nuclear division (mid-to-late schizogony), *Pb*Cen-4-GFP mainly localizes as a dot close to 1 nucleus (Fig 4B–4I and 4B’–4C’ and 4D’–4I’). This represents a single CP that starts its duplication and movement to the opposite part of the duplicating nucleus (Fig 4B–4D and 4B’–4C’ and 4D’–4I’) to finally result in 2 nuclei, each having again 1 CP (Fig 4E). One of the divided nuclei duplicates its CP (upper nucleus) while the second does not (lower nucleus) showing the asynchronicity of *Plasmodium* schizogony (Fig 4F). Then, start the S/M alternating phases that are repeated (Fig 4E–4I and 4D’–4I’) until the last synchronous round of replication finally completes merogony (Fig 4J and 4J’). However, the fate of the nuclear envelope during the asynchronous schizogony and karyokinesis in *Plasmodium* LS remains yet elusive in comparison to ABS [66].

CPs also appear to develop during parasite sporogony within the mosquito as *Pb*Cen-4-GFP clearly localizes to defined spots and has been confirmed in *P*. *falciparum* ABS, suggesting a shared mechanism between the different proliferative life cycle stages [8,65]. *Plasmodium* encodes 3 other centrins, namely Cen-1, Cen-2, and Cen-3, and it has been shown that the 4 centrins form a complex during early blood stage schizont. As Cen-1 to Cen-3 are known to be blood-stage essentials, their function is most likely not redundant; therefore, if Cen-1, Cen-2, or Cen-3 are individually deleted, the rest of the complex might not be fully functional, which could explain their essential role within *Plasmodium*. Whether centrins are implicated in LS CP biology by forming a complex remains to be investigated [8,42].

Although the actual mechanism of nuclear division might be quite similar in the different proliferative parasite stages, there are also distinct features of the LS, in particular, its extraordinary number of progeny (Fig 1). Molecules and mechanisms that have been reported to affect LS development will be discussed in the following section.

#### 2.1.3 LS-specific protein expression

Among the proteins that are specifically expressed during LS development is the PVM-protein UIS4. Although *uis4*-deficient parasites showed an early arrest in LS development [67], it is unlikely that UIS4 is directly involved in the cell division process. It is more apparent that UIS4 is required for the maintenance of PVM function and thus only indirectly affects LS parasite development.

This might be different for 2 other LS-specific proteins; the sporozoite surface protein essential for liver stage development (SPELD) and LISP2 (Table 1 and Fig 2F). Parasites deficient for each of the corresponding genes show an arrest during mid-to-late LS development [68,69]. SPELD localizes to the salivary gland sporozoite and EEF membrane in *Plasmodium* and is involved in merogony as *speld*-deleted parasites arrest at 36 hpi in vitro [69]. Microarray analysis of this mutant revealed a down-regulation of several LS-specific mRNAs including *uis4*, *lisp2*, and various genes involved in cell cycle and transcription [69]. A comparable phenotype has been observed for *lisp2*-deficient parasites that show an aberrant nuclear division and fail to complete merogony (Fig 2F) [68]. Although it has been shown that LISP2 is exported from the PVM to the host cell cytosol by secretory vesicles surrounded by LISP1 (a PVM protein) [68], it is likely that it has additional functions in the parasite including progress through development, as LISP2 also localizes in the parasite cytoplasm [68–70].

*Plasmodium* possesses an RBP that is an ortholog of the Meiosis inhibited 2 protein and is named PlasMei2. It is known to be required for initiating pre-meiotic DNA synthesis and chromatin reorganization during meiosis in *Schizosaccharomyces pombe* and plants, respectively [71–73]. In *P*. *yoelii* LS parasites, PlasMei2 has a cytosolic localization and presents a granular localization pattern similar to P-bodies in ABS, gametocytes, and sporozoites [37]. *PyPlasMei2*-knockout parasites were not affected in growth during early schizogony in vivo but in late schizogony, mutant parasites showed abnormal accumulation of DNA centers and a defect in DNA segregation (Table 1 and Fig 2E) [37]. *PyPlasMei2*-knockout parasites did not develop into the cytomere stage and therefore no infectious exoerythrocytic merozoites were formed [37]. Importantly, a similar phenotype was observed in *Pf*PlasMei2-deleted *P*. *falciparum* parasites [49] highlighting a crucial and conserved role of PlasMei2 for *Plasmodium* LS schizogony. *Pf*PlasMei2-deficient parasites are therefore considered as potential genetically attenuated parasites (GAPs) for human vaccination trials [49,74].

*P*. *berghei* LS parasites deficient in the LS AP2 transcriptional factor (AP2-L) known to control the expression of early LS genes also showed a nuclear division arrest phenotype (Fig 2E). This is consistent with the finding that *ap2-l* deletion results in the down-regulation of gene expression of *uis4*, *lisp1*, *lisp2*, notably all genes known to be involved in LS development [75].

Investigation of RNA-binding partners of PlasMei2 might help in answering the question as to whether LS schizogony could be regulated in part by RBP. Isolating protein-RNA complexes from parasite LS have been hampered due to the low sporozoite infection rate of hepatocytes; however, more sensitive mass spectrometry technologies could help to provide insight into LS schizogony regulation.

### 2.2 Organellar biogenesis and segregation

#### 2.2.1 The parasite mitochondrion and apicoplast segregation

In parallel to karyokinesis, the parasite expands its organelles including its mitochondrion and apicoplast to finally segregate and distribute them to individual merozoites. When a *Plasmodium* sporozoite invades a hepatocyte, it contains a single mitochondrion and apicoplast [5]. At around 24 hpi, when the parasite begins karyokinesis, the 2 organelles already appear as branched structures and this network grows in parallel to nuclear division but is not determined by it (Fig 2D) [6]. Similar to what has been shown for *Plasmodium* ABS schizogony [76], during the LS both the mitochondrion and the apicoplast do not perform binary fission after each nuclear division but instead grow to a large and branched structure [5,6]. The main difference between ABS and LS schizogony is that organelle expansion happens on a much greater scale during LS development and produces up to tens of thousands of merozoites as the erythrocytic schizogony produces up to 16 to 32 (Figs 1 and 2D–2H) [6].

During the cytomere stage, the mitochondrion lies within the spheres of membrane-encased groups of nuclei in clumped assemblies that remain as a single structure. The apicoplast, on the other hand, primarily lies between the nuclei and the surrounding plasma membrane (Fig 2E–2G). Later, the apicoplast starts to form regular constrictions and soon after, fission of the organelle occurs. In parallel, it appears that the mitochondrion develops finger-like structures pointing towards the aligned nuclei where fission of the mitochondrion occurs just prior cytokinesis (formation of single daughter merozoites) (Fig 2G and 2H) [5,6]. The ordered fission of the organelle networks suggests that fission machinery might only be present at a specific time point; however, to date, the crucial members of the mitochondrial (and apicoplast) fission machinery were not identified [76,77]. There are 2 dynamin orthologues present in the *Plasmodium* genome, Dyn1 and Dyn2 which potentially could perform such a function but they were found to exert functions other than mitochondrial fission [78,79].

Another protein that is closely associated in apicoplast biogenesis and segregation is *Plasmodium* autophagy-related protein 8 (ATG8). In contrast to mammalian or yeast ATG8, *Plasmodium* ATG8 is essential for parasite survival even under normal growing conditions as it is critical for the apicoplast inheritance into daughter parasites (Fig 2G) [80]. In *Pb*ATG8-overexpressing parasites, apicoplast redistribution between nascent liver merozoites is also impaired, demonstrating the importance of proper regulation of ATG8 protein expression [81]. *Plasmodium* ATG8 was shown not to be involved in parasite autophagy as it localized to the apicoplast throughout LS development, and even during parasite cell death it did not associate with autophagosomes [40]. This is not surprising as *Plasmodium* ATG8 lack the typical N-terminal processing known for all ATG8 homologues involved in autophagy. The association of ATG8 with the apicoplast through the whole LS development suggests a role in the organelle’s maintenance and expansion during intra-hepatic development. Interestingly, during parasite replication in hepatocytes, the association of *Pb*ATG8 with the apicoplast increases as this organelle expands in size, again, pointing towards a role in the late phases of apicoplast proliferation [82].

During ABS development, a close contact between mitochondrion and apicoplast was reported, and it is believed that this interaction is needed for exchange of metabolites for heme biosynthesis [4,83–85]. However, during LS schizogony, the association of the 2 organelles was less obvious until shortly before their division. After the fission of the apicoplast, the 2 organelles are then in close contact. Whether a physical interaction between these 2 organelles during LS exists and whether this is used to exchange metabolites remains to be elucidated [6].

Taking into account the immense growth of the mitochondrion, apicoplast, and PPM, it is obvious that the parasite requires a large lipid supply for the membranes of expanding organelles and future merozoites. Such an extensive lipid requirement in a relatively short time could partly explain why the fatty acid biosynthesis pathway is essential during exoerythrocytic development but is dispensable during the parasite’s development in the blood and for *P*. *berghei* in the mosquito [86,87]. Interestingly, for *P*. *falciparum*, the situation is different in that fatty acid biosynthesis is already essential during the oocyst formation in mosquitoes [88]. One reason could be that *P*. *falciparum* sporogony in mosquitoes is considerably faster than for rodent parasites.

#### 2.2.2 The needy time: The high demand of lipids for exoerythrocytic merozoite formation

*P*. *berghei* LS produces up to several tens of thousands of merozoites in comparison to only tens in the ABS [89]. One reason for parasite LS to produce more merozoites might be the hepatocyte size. In comparison, parasite ABS have a restricted space within the erythrocyte. Uninfected erythrocytes have a volume of approximately 90 μm^3^ [90] and although parasites growing in erythrocytes can increase the volume of the host cell, it cannot exceed beyond 150 μm^3^ [90]. On the other hand, hepatocytes have a volume of about 10,000 μm^3^ but at its largest, the LS parasite can reach up to 50,000 μm^3^ [36], 300 times larger than the volume of an ABS parasite, which also correlates with the number of merozoites produced (8 to 32 in erythrocyte versus 10,000 to 100,000 in hepatocyte, depending on the *Plasmodium* species) (Fig 1). Therefore, *Plasmodium* parasites might be able to sense the host cell membrane as a physical barrier or may simply be restricted by the supply of nutrients. The massive growth of LS parasites raises the question as to how the parasite acquires enough phospholipids to support the expansion of its plasma membrane. Different scenarios can be exploited to respond to the tremendous lipid demand of the rapidly growing parasite.

First, *Plasmodium* parasites are known to scavenge lipids from the host cell during intraerythrocytic development [91]. During LS development, it was shown that the PVM protein UIS3 interacts with the host cell liver fatty acid-binding protein (L-FABP) and this interaction has been speculated to be a way for the parasite to uptake lipids from its host cell [92,93]. Another scenario could be that the lipid stock might stem from host cell late endosomes (LE) and/or lysosomes that fuse with the PVM [94,95] as cholesterol in the PVM, for example, was shown to derive from late endosomes and lysosomes and blocking cholesterol sequestration from LE and lysosomes greatly impaired LS development [96]. Finally, LS parasites are known to closely interact with host cell mitochondria, endoplasmic reticulum (ER) and Golgi [97–99], and membrane contact sites between the host mitochondria and the parasite PVM have been observed, suggesting lipid scavenging by the parasite from this organelle [97].

In addition to scavenging lipids from the host cell, the parasite requires a de novo fatty acid synthesis pathway to complete its development within the hepatocyte via a bacterial-like type II pathway called FASII (reviewed in [100,101]). The FASII pathway is located in the apicoplast where acetyl-CoA acts as the main precursor for fatty acid synthesis [101]. Acetyl-CoA is synthesized from pyruvate via the pyruvate dehydrogenase complex (PDH) or from acetate via the acetyl-CoA synthetase (ACS) [100,102]. Biosynthesis of fatty acids is mediated by 4 enzymes in the elongation cycle of FASII. The 4 enzymes in this cycle are β-ketoacyl-acyl carrier protein (ACP) synthase I/II (FabB/F), β-ketoacyl ACP reductase (FabG), β-hydroxyacyl-ACP dehydratase (FabZ), and enoyl-ACP reductase (FabI) [10,102]. Knocking out FabB/F and FabZ in *P*. *yoelii* prevented the parasite LS from forming blood-infective merozoites, and *P*. *berghei* parasites deleted in FabI and FabG were strongly impaired in LS development, resulting in a severe delay in the pre-patent period in mice (Fig 2F) [10,86,103]. More recently, single knockout parasites of genes participating in the FASII process such as a biotin-acetyl-CoA-carboxylase (HCSI), dihydrolipoamide acyltransferase (PDH-E2), malonyl CoA-ACP transacylase (FabD), β-ketoacyl-ACP synthase III (FabH), and lipoyl synthase (LipA) showed a drastic defect during late LS development in vitro and a delay in the pre-patent period when mutant sporozoites were injected into mice [10]. Both studies demonstrated that in *P*. *berghei*, FASII enzymes are dispensable for mosquito colonization, oocyst development, and sporozoite formation. This is highlighting the importance of these proteins during *P*. *berghei* LS merogony, when more lipids are needed in a short time (Fig 2F–2H). Since early LS parasites (trophozoite, early schizonts) require less lipids, they develop rather normally in the absence of these enzymes [10,86,103]. Fatty acids produced by FASII in the apicoplast contain 10 to 14 carbons. To further elongate fatty acids for incorporation into phospholipids, they are transported to the ER where fatty acid elongases (ELO-A, ELO-B, and ELO-C) present in the ER synthesize long-chain fatty acids. It is known that a ketoacyl-CoA reductase (KCR), a hydroxyacyl-CoA dehydratase (DEH), and an enoyl-CoA reductase (ECR) are required for the final elongation step [102]. Moreover, an NADH-cytochrome b5 reductase (CBR), which functions as a fatty acyl desaturase, introduces double bonds to generate unsaturated fatty acids before being integrated into phosphoglycerolipids and cellular structures [102,104]. ELO-A, KCR, and CBR were shown to be important for LS growth and formation of detached cells in vitro [10]. *Plasmodium* LS have an ER with a vast network of branches and large accumulation assemblies [7]. These accumulations were shown to be stacks of membrane material that might act as phospholipid storage units [7] and once the parasite forms PPM invaginations during the cytomere stage, the lipid storage in the ER can be tapped. Providing enough phospholipids for PPM biogenesis is, however, not the only reason for the essentiality of the FASII pathway. Other organelles such as the mitochondrion, the apicoplast, the Golgi apparatus, secretory organelles (micronemes, rhoptries), and the inner membrane complex (IMC) also need lipids to be elongated or formed de novo. Moreover, many membrane proteins in *Plasmodium*, such as merozoite surface protein 1 (MSP1), have glycosylphosphatidylinositol (GPI) anchors. MSP1 is an important surface protein of merozoites and makes up, together with MSP2, around two-thirds of all GPI-anchored proteins in merozoites [105]. FASII might therefore play an important role in supplying enough fatty acids for GPI biogenesis. FASII could also be responsible for supplying specific fatty acids that are only used in LS merozoites [102]. GPI anchors are generated in the ER and remodeled until the protein is incorporated into the plasma membrane [106]. It is plausible that GPI-anchored proteins during the LS need specific lipids that can only be produced by FASII [102].

As previously mentioned, apicoplast and mitochondrion segregation has been extensively investigated within the *Plasmodium* LS development but how the Golgi apparatus, the secretory organelles, and the IMC are formed and are segregated during schizogony/merogony in the parasite liver phase remains largely unknown. The merozoite organizing protein of *P*. *falciparum* (*Pf*MOP) is involved in IMC formation leading to a complete segmentation of daughter merozoites through plasma membrane budding in *Pf*ABS [107]. Conditional disruption of *Pf*MOP in blood stage parasites showed a defect in parasite segmentation. *Pf*MOP is not essential for blood stage progression as the mutant was able to form infectious merozoites. Interestingly, it remains essential for transmission as *Pf*MOP-deficient gametocytes were not viable [107]. As *mop* mRNA of *P*. *berghei* is expressed by LS schizonts [9], it would now be important to investigate whether *Pf*MOP is also essential for this parasite stage.

The use of specific antibodies targeting proteins localized at the Golgi apparatus, secretory organelles and IMC, or parasite lines expressing tagged marker proteins could help in deciphering the distribution of these vital organelles during cytokinesis, as has been shown for the apicoplast, the mitochondria, and the nuclei [5,6].

To manage the extraordinary PPM demand during merogony, *Plasmodium* LS parasites are also able to shuffle lipids from the parasite ER to the PPM at membrane contact sites [56]. To control the correct fusion of these membranes of different organelles, *Plasmodium* SNARE proteins are thought to be involved. Although the parasite genome encodes several SNARE proteins, their role in ER-PPM contact sites needs to be confirmed [56].

Once karyokinesis and cytoplasmic division at the end of LS is completed, newly formed merozoites are physically separated and egress from the hepatocyte is prepared. Even though the molecular mechanism of merozoite formation and egress is not fully understood for LS parasites, some interesting aspects will be discussed in the following section.

### 2.3 From the cytomere stage to individual merozoite

During the cytomere stage, *Plasmodium* parasites complete merogony by segmenting the newly formed merozoite and surrounding it by the newly formed PPM. This process can be considered as a special form of cytokinesis (Fig 2F–2I). In most other eukaryotic cells, cytokinesis begins during late mitosis (anaphase) by the formation of a contractile ring around the equator of the cell just beneath the plasma membrane. When the contractile ring shrinks, the plasma membrane is pinched off until the 2 daughter cells have physically separated [108].

In *Plasmodium* ABS, it has long been speculated that the final round of nuclear division occurs synchronously and simultaneously with cytokinesis [1]. Recent visualization of the 3D ultrastructure of *Plasmodium* parasites by Focused Ion Beam-Scanning Electron Microscopy (FIB-SEM) of *Pf*ABS segmentation, however, suggested that the last round of nuclear division was asynchronous [109]. In contrast, the final steps of LS karyokinesis and merogony in *Plasmodium* LS were rather synchronous [6,8,56]. The difference of these observations might simply be technical as live imaging was used for the LS. As FIB-SEM has already been used to show the endopolygeny-like nuclear architecture of the mosquito oocyst stage of *Plasmodium cynomolgi* [110], it will also likely be useful for analyzing nuclear division and cytokinesis during the cytomere LS stage.

At a molecular level, little is known about LS cytokinesis. In *P*. *berghei*, a protein that might be involved is the *Plasmodium*-specific apicoplast protein important for liver merozoite formation (PALM), as in its absence, the final step of merozoite segregation is aborted (Table 1) [111]. Other proteins that might be involved in cytokinesis are cyclin 1 (Cyc1) and coordinator of nascent cell detachment (CINCH), which are expressed in *P*. *falciparum* and *P*. *berghei* ABS and LS [9,47,112]. Surprisingly, deletion of Cyc1 in *P*. *falciparum* did not affect ABS nuclear division but showed a failure in enclosure of the nascent merozoite, leaving the cytoplasm of the forming merozoites connected [47]. A similar phenotype has been observed upon deletion of *Pfcinch*. CINCH-deficient *P*. *falciparum* ABS parasites have a defect in the generation of daughter cells [112]. In both cases, *Plasmodium* mutants give rise to aneuploid merozoites having varied cellular and nuclear size. Interestingly, despite the severe morphological defects, these mutants are liberated from their host cell suggesting that there is no direct link between fully completed cytokinesis and parasite egress. Both *cyc1* and *cinch* transcripts are detected during LS schizogony and merogony and show a similar expression as in ABS schizogony. However, whether they are involved in LS cytokinesis remains to be proven.

### Concluding remarks and open questions

This review summarizes our knowledge on *Plasmodium* LS cell division and development. Although we are beginning to understand some molecular details of these processes, we are far from having a complete picture. One of the main questions is whether the processes acting in ABS are also employed for LS cell division. Both life cycle stages result in the formation of merozoites infectious toward red blood cell, but in this review, we have pointed out the important differences between both stages. Still, we referred a lot to what is already known for *P*. *falciparum* ABS to fill the larger gaps in our knowledge of LS cell division, but this urgently needs experimental confirmation. This is not an easy task as a straight knockout strategy is not possible if the corresponding gene is essential for blood stage development, which is the stage used for genetic manipulation. The other problem is the low infection rate of *Plasmodium* sporozoites that makes protein purification difficult. Co-immunoprecipitations combined with highly sensitive mass spectrometry might be the solution to study protein–protein interaction in *P*. *berghei* LS parasites [11,113].

To date, it is not clear how conserved the mechanism of cell division is among *Plasmodium* species. To understand LS biology, we mainly refer to the rodent species as they are standardized models that are relatively easy to maintain in the laboratory. However, the main goal must be to extrapolate the knowledge to *P*. *falciparum* LS. Recent success in cultivating axenic *P*. *falciparum* mosquito stages and the successful generation of salivary gland sporozoites to infect human hepatocytes or humanized mice will have a big impact in malaria research in this respect [114].

An interesting aspect that has not been explored in this review and remains fundamental is the asexual sporogony event that occurs during the mosquito stage and shows some similarities to asexual schizogony in the vertebrate host. Even though the insect and mammal environments are distinct, and the parasites reside extracellularly in oocysts during the mosquito stage, the obligation for *Plasmodium* to produce a high number of daughter cells remains during sporogony. A shared feature in sporogony and schizogony is the formation of a CP during the karyokinesis process. This has been observed by following the expression of *Pb*Cen-4-GFP through liver and mosquito stages. Also, the formation of merozoites and sporozoites appears to follow similar routes, as PPM invagination has been observed for both events [8,56].

Another topic of *Plasmodium* LS development that is yet to be explored is cell cycle control of dormant human *P*. *vivax* and the simian *P*. *cynomolgi* parasites. *Plasmodium* LS entering dormancy implies that the sporozoite is not enrolled in a productive schizogony. They rather remain small but still grow at a reduced rate. This single-nucleated dormant form is also called hypnozoite (Fig 2C’) (reviewed in [115]). These dormant parasites can remain quiescent within the host liver for many years and eventually relapse by forming schizonts and BS infective merozoites. Reasons for the commitment of sporozoite into hypnozoite remain largely unknown [115]. It has been suggested that the transcription factor AP2-Q is up-regulated in quiescent hypnozoite forms [116] but others could not confirm this [117,118]. Still, transcription factors might be involved in maintaining the dormant stage including other AP2 members or non-AP2 transcription factors like the Myb protein [115,119]. It is still not entirely clear whether a proportion of sporozoites are already committed to becoming dormant. Single sporozoite mRNA sequencing might help to identify differences in *P*. *vivax* or *P*. *cynomolgi* sporozoite populations [120]. Another option is that dormancy is rather defined by the host cell environment. Stress and intracellular signaling resulting in autophagy-like responses [121] are factors that vary in different host hepatocytes and trigger dormancy [122]. In spite of a general metabolic shutdown, hypnozoites pursue the expression of pathways likely involved in the prolongation of dormancy including ATP homeostasis and chromatin maintenance. Concerning the cell cycle, we could consider hypnozoites as being in a G0 state where DNA synthesis and expression of some of cell cycle effectors as cyclin-CDKs are down-regulated as observed in *Pf*ABS under artemisinin-drug pressure [123].

Equally important in this respect is the question as to how hypnozoites relapse. So far, no relapsing signal or a cell cycle signal has been identified, but there is some indirect evidence for intrinsic factors like a genetically defined internal clock [119,124]. Still, as yet unknown extrinsic factors may also contribute [125]. Research in this direction is gearing up and exciting new findings can be expected in the coming years that might help to identify desperately needed new strategies against dormant LS parasites. Together, LS cell division remains a very important topic in malaria research and will be key in the attempt to eradicate malaria.
